# Microbes as Resources to Remove PPCPs and Improve Water Quality

**DOI:** 10.1111/1751-7915.70084

**Published:** 2025-01-27

**Authors:** Francesca Demaria, Marcel Suleiman, Philippe Corvini, Pilar Junier

**Affiliations:** ^1^ Institute for Chemistry and Bioanalytics University of Applied Sciences and Arts Northwestern Muttenz Switzerland; ^2^ Laboratory of Microbiology, Institute of Biology University of Neuchatel Neuchatel Switzerland

## Abstract

The inadequate removal of pharmaceuticals and personal care products (PPCPs) by traditional wastewater treatment plants (WWTPs) poses a significant environmental and public health challenge. Residual PPCPs find their way into aquatic ecosystems, leading to bioaccumulation in aquatic biota, the dissemination of antibiotic resistance genes (ARGs), and contamination of both water sources and vegetables. These persistent pollutants can have negative effects on human health, ranging from antibiotic resistance development to endocrine disruption. To mitigate these risks, there is a growing interest in exploiting microorganisms and their enzymes for bioremediation purposes. By harnessing the metabolic capabilities of microbial communities, PPCPs can be efficiently degraded, transformed, or sequestered in water systems. Additionally, microbial communities exhibit remarkable adaptability and resilience to diverse PPCP contaminants, further underscoring their potential as sustainable and cost‐effective solutions for water treatment. This review explores the promise of microbial bioremediation as an approach to addressing the complex challenges posed by persistent PPCP contamination, emphasising its potential to safeguard both environmental integrity and human well‐being.

## Graphical abstract

This review explores the promise of microbial bioremediation as an approach to addressing the complex challenges posed by persistent pharmaceuticals and personal care products contamination, emphasising its potential to safeguard both environmental integrity and human well‐being.

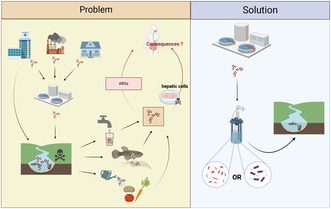

**Abbreviations**
AIAPs: analgesic and anti‐inflammatory pharmaceuticals
API: active pharmaceutical ingredient
ARGs: antimicrobial resistance genes
CNS: central nervous system
EC_50_
: Half maximal effective concentration
EE2: ethinylestradiol
NSAIDs: non‐steroidal anti‐inflammatory drugs
PPCPs: pharmaceuticals and personal care products
WWTP: wastewater treatment plant

## 1 Introduction

The pervasive presence of pharmaceuticals and personal care products (PPCPs) in water represents a pressing concern, with potential health implications for both ecosystems and humans. This new type of pollution, also called micropollutant pollution, is the result of extensive production and consumption of PPCPs (Persson et al. 2013; Steffen et al. 2015). Even though the direct effects of PPCPs in the environment are still under investigation, the consequences of the release of these chemicals are very likely negative. Some PPCPs are persistent and have a likely disruptive effect on biodiversity (Madsen, Quiblier, and United Nations Environment Programme 2013; Persson et al. 2013). Multiple scientific developments aim to help address the issue of environmental micropollutant pollution. The increase in sensitivity of analytical methods for the detection of pollutants and degradation products is helping to reveal the extent of the problem (Nikolaou 2013). Furthermore, wastewater treatment plant (WWTP) operators are implementing new technologies for micropollutant removal (Erythropel et al. 2018; Nikolaou 2013; Persson et al. 2013). Amongst them, bio‐based solutions are promising as a complementary advanced method for PPCP removal from water (Shah 2020).

This review aims to shed light on biotechnological solutions for improving pharmaceutical removal in WWTP to limit the adverse effects of PPCPs on the health of humans and ecosystems in a sustainable manner. Bio‐based solutions align with the UN's sustainable development goals (SDG), ensuring societal health and well‐being (SDG 3) while also advancing water sanitation goals (SDG 6) (Shah 2020). This is supported by its economic feasibility and environmental sustainability compared to alternative advanced techniques such as electrochemical treatment, ion exchange, or filtration (Milić, Avdalović, and Knudsen 2024).

## 2 PCPPs in the Environment

Active pharmaceutical ingredients (APIs) (Pot et al. 2022) is a category of PPCPs found in water sources all over the world because they are often hydrophilic and not volatile. API concentrations in the environment range from μg/L to ng/L (Wang et al. 2021; Kallenborn et al. 2008; Kmmerer 2010; Pot et al. 2022). A study in 2021 investigated surface waters across 24 countries covering a broad spectrum of climatic and environmental conditions (Pot et al. 2022). The results demonstrated that 14 APIs were found in all continents (excluding Antarctica). The primary source of APIs are industrial production sites (Larsson, de Pedro, and Paxeus 2007). Hospitals also represent a common source of APIs, when wastewater containing high concentration of PPCPs is released without pre‐treatment (Gómez et al. 2006). However, outpatient consumption and inadequate disposal represent one of the most challenging sources to tackle. According to surveys conducted in 2021 in the USA, most of the responders (up to 88%) have unused PPCPs at home, and 55% of those responders dispose PPCPs in the toilet instead of the apposite sites (Caban and Stepnowski 2021). The problem is aggravated by an increase each year in access through excessive prescriptions (Caban and Stepnowski 2021). The quantity of prescribed medications surged by up to 85% in 2019, in contrast to a 21% in population growth during the same period. The overuse of medications likely results in greater pollution from improper drug disposal and the release of partially metabolised substances (Caban and Stepnowski 2021). Moreover, the high use of PPCPs in agriculture and livestock farming also contributes to the problem (Rzymski, Drewek, and Klimaszyk 2017).

## 3 The Consequences of Chemical Pollution

The widespread use and inadequate disposal of PPCPs introduce a new dimension of pollution. PPCPs are already recognised as a threat to aquatic animals due to their impact on the endocrine and reproductive systems (Yuan et al. 2023). Some APIs exert their effect at very low concentrations, as is the case for instance of ethinylestradiol, which can have an impact on the feminization of *Pimephales promelas* at a concentration as low as 5–6 ng/L (Aus der Beek et al. 2016). Even though the concentration of individual PPCPs in environmental samples is often below the concentration that has negative consequences on 50% of the tested organism populations in laboratory tests (EC_50_), one needs to consider that in the environment multiple PPCPs may exert synergistic or antagonistic effects on aquatic life as well as potentially affect human health (la Farré et al. 2008; Schröder et al. 2016).

In addition, both known and unknown transformation products remain insufficiently studied even though they could be more harmful (De Boer et al. 2011). In a study performed at Cornell University in 2021, 23 out of 38 analysed xenobiotics were biotransformed into metabolites by wastewater treatment microbial communities (Rich, Zumstein, and Helbling 2022). Therefore, the real status of chemical pollution is likely more worrisome since most studies focus on the parent compounds while neglecting biotransformation products and metabolites (Aus der Beek et al. 2016; Dos S. Grignet et al. 2022; Rich, Zumstein, and Helbling 2022; Yan et al. 2023). The parent compound is found hydroxylated, such as atenolol, carbamazepine, or conjugates, such as ibuprofen and naproxen (Kasprzyk‐Hordern, Dinsdale, and Guwy 2008). In some cases, biotransformation products and metabolites are more toxic than the parent compounds, such as in the case of 11‐dihydroxy‐carbamazepine, which was found to be 10 times more toxic than carbamazepine in bioassays with *Chironomus tiparius* (Yang et al. 2020). Furthermore, some PPCPs and their intermediates are prone to be bioaccumulated resulting in some cases in biomagnification in fish and mussels, and ultimately, human exposure (Yan et al. 2023). For example, methylated diclofenac is more hydrophobic, therefore more prone to accumulate in tissues; therefore, diclofenac methyl ester is found in higher concentration than diclofenac in two crustacean species, *Hyalella azteca* and *Gammarus pulex* (Maculewicz et al. 2022). Moreover, acetylated SMX and ciprofloxacin are found more in see cucumber upon exposure to the parent compound (Maculewicz et al. 2022). Another exposure pathway corresponds to irrigation of vegetables with reused wastewater and drinking water containing PPCPs. Some studies have confirmed the uptake of micropollutants by carrots, potatoes, and cabbages, with contaminants persisting even after peeling (Goldstein, Shenker, and Chefetz 2014; Herklotz et al. 2010; Malchi et al. 2014).

Concerning a direct impact on human health, the risks assessment of PPCPs in water is still under debate (Zenker et al. 2014). The contamination of drinking water is poorly examined due to analytical detection challenges and the associated increase in costs and, accordingly, the data to draw meaningful conclusions is still missing. Nevertheless, a study on the growth of hepatic cells in presence of a cocktail of 12 pharmaceuticals at ng/L concentration indicated a 10%–30% growth inhibition (Pomati et al. 2006). Furthermore, in another study it was demonstrated that oestrogen exposure at a concentration of 2.5 μg/kg affected fertility in a murine model (Newbold 1995). These studies highlight the potential consequences on health.

Finally, the presence of PPCPs in water contributes to the emergence and spreading of antibiotic resistance genes (ARGs) (Larsson and Flach 2022). The exposure of pathogenic microorganisms to antibiotics and other PPCPs at low concentrations facilitates the spreading of ARGs. For instance, the mammalian pathogen *Helicobacter pylory* exposed to 16 μg/L of ciprofloxacin increased by five–six‐fold its DNA repairing mechanism rate. This increased the integration of external DNA in the bacterial genome and thus the chance of acquiring ARGs (Dorer, Fero, and Salama 2010). Additionally, it was demonstrated under laboratory conditions by Wang et al. that oxidative stress with reactive oxygen species formation is already provoked at 0.5 mg/L of some PPCPs (diclofenac, ibuprofen, and naproxen to ß‐blockers‐ propranolol). This induces an increase of membrane permeability and bacteria transformation rate, enhancing by 40% the spreading of ARGs in ARG‐free microorganisms compared to the control unexposed to PPCPs (Wang et al. 2020). This phenomenon can also have indirect repercussions on human health (Tijani, Fatoba, and Petrik 2013).

## 4 Properties of PCPPs

This review focuses on eight categories of PPCPs: analgesic and anti‐inflammatory pharmaceuticals (AIAPs), beta‐blockers, central nervous system (CNS) stimulant, antihypertensives, xenoestrogen, antimicrobial, antibiotics, and psychiatric drug (Table 1).

**TABLE 1:** PPCPs commonly found in effluents of WWTP. All the reported values are the maximal concentration (if reported in the article) detected in treated effluents of WWTP.

Category	PPCPs	Concentration (ng/L)	Location	Reference	Structure
AIAPs ^a^	Acetaminophen	4300	Spain	Gómez et al. (2007)	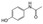
		112,780	USA	Kibuye et al. (2019)	
		62,000	Canada	Guerra et al. (2014)	
	Diclofenac	37,000 ± 2000	Nigeria	Ajibola et al. (2021)	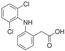
		1618 ± 18	Portugal	Salgado et al. (2010)	
		507 ± 5	Italy	Patrolecco et al. (2013)	
		561	New Zealand	Kumar, Sarmah, and Padhye (2019)	
		1805	United Kingdom	Kay et al. (2017)	
		2200	Spain	Gómez et al. (2007)	
		9700	South Africa	Madikizela and Chimuka (2017)	
	Naproxen	526 ± 12	Italy	Patrolecco et al. (2013)	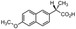
		34,718,990	USA	Kibuye et al. (2019)	
		14,400	South Africa	Patrolecco et al. (2013)	
	Ibuprofen	43,653 ± 54	Portugal	Salgado et al. (2010)	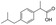
		62,000 ± 900	Nigeria	Ajibola et al. (2021)	
		28,000	Spain	Gómez et al. (2007)	
		1003 ± 9	Italy	Patrolecco et al. (2013)	
		2972	United Kingdom	Roberts and Thomas (2006)	
		14,231	United Kingdom	Kay et al. (2017)	
		19,200	South Africa	Patrolecco et al. (2013)	
		4700	Canada	Guerra et al. (2014)	
ß‐blockers	Atenolol	1297 ± 14	Portugal	Salgado et al. (2010)	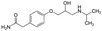
		611	New Zealand	Kumar, Sarmah, and Padhye (2019)	
		6100	Hong Kong	Ruan et al. (2019)	
CNS^b^ stimulant	Caffeine	4392 ± 22	Portugal	Salgado et al. (2010)	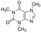
		44,300	Spain	Gómez et al. (2007)	
		535.3 ± 97.2	USA	Bartelt‐Hunt et al. (2009)	
Antihypertensives	Enalapril	19,888 ± 22	Portugal	Salgado et al. (2010)	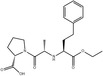
		55	Sweden	Lavén et al. (2009)	
Xenoestrogen	Ethinylestradiol	137 ± 24	China	Lei et al. (2020)	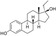
		187	Argentina	Valdés et al. (2015)	
		549 ± 13	China	He et al. (2013)	
		340	Tunisia	Belhaj et al. (2015)	
Antimicrobial agent	Triclosan	33	New Zealand	Kumar, Sarmah, and Padhye (2019)	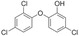
		400	Spain	Gómez et al. (2007)	
		490	Canada	Guerra et al. (2014)	
		1751	India	Mohan and Balakrishnan (2019)	
		13,000	South Africa	Lehutso, Daso, and Okonkwo (2017)	
		430 ± 22	Switzerland	McAvoy et al. (2002)	
Antibiotics	Sulfamethoxazole	141.4 ± 22.3	USA	Bartelt‐Hunt et al. (2009)	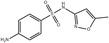
		317,000	USA	Kibuye et al. (2019)	
		1800	Canada	Guerra et al. (2014)	
	Ciprofloxacin	31,000,000	India	Larsson, de Pedro, and Paxeus (2007)	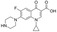
		473	Canada	Guerra et al. (2014)	
	Trimethoprim	322	New Zealand	Kumar, Sarmah, and Padhye (2019)	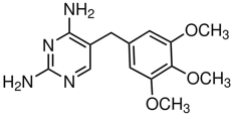
		532	United Kingdom	Roberts and Thomas (2006)	
		580	Finland	Kortesmäki et al. (2020)	
		78 ± 9	Canada	Guerra et al. (2014)	
Psychiatric drug	Carbamazepine	793	Italy	Patrolecco et al. (2013)	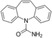
		230	New Zealand	Kumar, Sarmah, and Padhye (2019)	
		119.5 ± 15.6	Spain	Gómez et al. (2007)	

^a^AIAPs stands for analgesic and anti‐inflammatory pharmaceuticals, ^b^CNS stands for central nervous system.

### 4.1 AIAPs

The majority of the AIAPs are grouped under the category of non‐steroidal anti‐inflammatory drugs (NSAIDs). One of the mild AIAPs that does not fall into the category of NSAIDs is acetaminophen. Acetaminophen, diclofenac, ibuprofen, and naproxen inhibit the action of prostaglandins that trigger an inflammatory response, resulting in a pain release action (Brunton, Chabner, and Knollmann 2011). These compounds are widely used all over the world and, in most cases, can be bought over the counter (Dos S. Grignet et al. 2022). For this reason, they are detected in mg/L range in aquatic ecosystems, especially in the proximity of hospitals and households (Aydin, Mehmet Aydin, and Ulvi 2019). Moreover, most AIAPs do not sorb to activated sludge owing to their chemical composition, which in conjunction with their low biodegradability, leads to poor removal efficiency (Aydin, Mehmet Aydin, and Ulvi 2019). Samples collected in the effluent of a private hospital in Almería showed that ibuprofen, diclofenac, and acetaminophen could be detected in concentrations ranging between 0.1 and 100 μg/L (Gómez et al. 2006). At a concentration of μg/L, these three compounds represent a high/moderate toxicity risk for representative taxa—fish, algae, and Daphnia (Aydin, Mehmet Aydin, and Ulvi 2019). For example, diclofenac and ibuprofen were of concern for the reproductive system of the fish *Hyalella azteca* (Zenker et al. 2014).

Acetaminophen is less recalcitrant to biodegradation than other analgesics, but due to its large consumption, it is one of the most frequently detected AIAPs in water bodies (Dos S. Grignet et al. 2022; Wu, Zhang, and Chen 2012). Acetaminophen concentrations of WWTP effluents in Canada, Spain, and the USA were found to be in the range of μg/L (Gómez et al. 2007; Guerra et al. 2014; Kibuye et al. 2019). Moreover, ibuprofen, diclofenac, and naproxen were found in Mbokodweni River of South Africa in concentrations in the range of μg/L due to a limited removal efficiency of WWTPs (Madikizela and Chimuka 2017). In a WWTP effluent in Pennsylvania, USA, naproxen has been detected in a concentration that is even higher—in the mg/L range (Aydin, Mehmet Aydin, and Ulvi 2019; Kibuye et al. 2019).

### 4.2 ß‐Blockers

ß‐blockers act as competitors of adrenaline, interacting with the G‐protein‐coupled (GPCR) family of adrenoreceptors; this leads to a decrease of the heartbeat rate. ß‐blockers are used during heart failure, angina pectoris, and arrhythmia (Gorre and Vandekerckhove 2010; Martínez‐Milla et al. 2019). Amongst them, atenolol is the most used clinically (Carlberg, Samuelsson, and Lindholm 2004). This compound has been found in a concentration of μg/L in water basins all over the world. Atenolol was detected at the maximum concentration of 6.1 μg/L in a WWTP effluent in Hong Kong (Carlberg, Samuelsson, and Lindholm 2004; Ruan et al. 2019; Yi et al. 2020). It is worth mentioning that degradation intermediates generated by atenolol degradation are more toxic than the parent compound to model organisms for ecotoxicological analysis, such as fish or Daphnia. Atenolol is also classified as a high‐risk compound for algae (Yi et al. 2020). Moreover, the target of ß‐blockers (G‐protein) has homologous G‐protein in fish and could cause harm in marine environments (Yi et al. 2020), and these compounds are known to be biomagnified in mussel gills (Zenker et al. 2014).

### 4.3 CNS Stimulant

Caffeine is a purine base that acts as a stimulant for the central nervous system. It is the antagonist of the adenosine receptor and stimulates hormones such as epinephrine, which increases cognitive functions (Fiani et al. 2021). Caffeine is detected with a frequency of 50% in 1052 river sampling sites across the world (Pot et al. 2022). It was detected in high concentrations in fresh water, according to a global study carried out worldwide in 2021 (Pot et al. 2022). The concentration of caffeine found in the environment reached 44 μg/L in a WWTP effluent in Spain, 120 μg/L in a raw wastewater sample, and 642 μg/L in WWTP influent in China (Gómez et al. 2007; Li et al. 2020). Caffeine induces oxidative stress and detoxification in the bivalve *Ruditapes philippinarum* even at a low concentration (0.1 μg/L) (Aguirre‐Martínez, DelValls, and Martín‐Díaz 2016).

### 4.4 Antihypertensives

Enalapril is one of the pharmaceuticals used in cases of hypertension and heart failure. It acts as an angiotensin inhibitor with a consequent reduction of angiotensin II and aldosterone that results in a reduction in blood pressure (Gomez, Cirillo, and Irvin 1985). Enalapril is highly used, but its removal is poorly studied. In Brazil, enalapril is provided free of charge for treating hypertension in the Brazilian Pharmacy Program, which results in high consumption (Cunha et al. 2023). Moreover, it was found in high concentrations in effluents in a study conducted in Portugal in 2010, with a concentration of 20 μg/L (Salgado et al. 2010).

### 4.5 Xenoestrogen

17‐α‐ethinylestradiol (EE2) is a synthetic oestrogen used as a contraceptive for humans. It falls in the category of endocrine‐disruptive chemicals (Caldwell et al. 2008; Siegenthaler et al. 2017). EE2 is of concern since it is frequently found in the effluents of WWTPs and can cause some abnormalities on the reproductive system of the aquatic biota, such as feminization and infertility (King et al. 2016; Tang, Liu, et al. 2021). Moreover, it is ubiquitous, and experts state that 65% of water is considered at risk concerning EE2 pollution (Tang, Liu, et al. 2021). In many cases, EE2 was detected in WWTP effluents in China in concentrations above the European good quality standard of water, with concentrations around 549 ng/L (He et al. 2013; Tang, Liu, et al. 2021). Moreover, the EE2 removal efficiency of WWTPs oscillates, and in some cases EE2 is not degraded at all (Tang, Liu, et al. 2021).

### 4.6 Antimicrobial Agents

Triclosan is a halogenated phenolic compound widely used for the elimination of bacteria and fungi. It disrupts proteins and fatty acid synthesis, causing cell death. It is vastly used in personal care products and disinfectants in Europe, America, and Asia. It is detected all over the world in WWTP effluents at concentrations around 500 ng/L (Canada, Spain, Switzerland), or even higher (i.e., 13 μg/L in South Africa) (Gómez et al. 2007; Guerra et al. 2014; Lehutso, Daso, and Okonkwo 2017; McAvoy et al. 2002). In some cases, in WWTP triclosan is methylated, generating a more lipophilic compound that ends up in water and is bioaccumulated in algae or tissues of fish, snails and whales (Dann and Hontela 2011; Yueh and Tukey 2016). In the presence of organic compounds in water, triclosan can undergo photocatalytic degradation that can lead to the production of highly toxic and persistent compounds, such as dioxins. Triclosan exerts adverse effects on mammals, and it has been shown to have a carcinogenic effect in mice (Yueh and Tukey 2016).

### 4.7 Antibiotics

Antibiotics are substances derived from natural products or synthetically produced that are used to prevent the spread of microbial infections. According to their mechanism of action and structure, antibiotics are classified in different groups (Hutchings, Truman, and Wilkinson 2019). Antibiotics are essential for the treatment of bacterial infections in humans and are extensively used all over the world. In 2019, it was estimated that 100,000–200,000 tons of antibiotics were consumed per year, globally (Omuferen, Maseko, and Olowoyo 2022). Their metabolization is generally very low (between 30% and 15%), resulting in the excretion of unchanged antibiotics in the environment (Khasawneh and Palaniandy 2021). The efficiency of antibiotics removal by wastewater treatment plants (WWTPs) varies depending on the treatment methods, time of year, and the specific pharmaceuticals present. Even after being removed from the water, antibiotics can accumulate in activated sludge and suspended solids, potentially re‐entering the environment later. Therefore, humans, marine biota, and microorganisms can be exposed to antibiotics for long periods of time (Khasawneh and Palaniandy 2021). The persistence of antibiotics in the environment is threatening for human health since it exerts a selective pressure on bacteria to develop resistance. The pollution by antibiotics favours the spreading of ARGs amongst microbial communities and is one of the main health concerns of modern societies (Feng, Huang, and Chen 2021; Hutchings, Truman, and Wilkinson 2019; Larsson and Flach 2022).

### 4.8 Psychiatric Drug

Carbamazepine is used to treat nerve pain and epilepsy, and it acts as an adenosine receptor inhibitor (Tolou‐Ghamari et al. 2013). It is persistent in WWTP effluents because removal efficiency in WWTPs fluctuates between 30% and 60% (Adeyanju et al. 2022). Therefore, carbamazepine is frequently detected in the environment. It was detected in 65% of a total of 1052 water sampling sites, reaching a concentration of 23 ng/L in drinking water (Feijoo, Kamali, and Dewil 2023). Moreover, carbamazepine has negative effects on marine biota since it affects the transmission of neurological signals. More studies are required to establish the consequences of environmental pollution by carbamazepine (Adeyanju et al. 2022; Batucan et al. 2022).

## 5 Role of WWTP Systems in the Removal of Chemical Pollutants

It is widely accepted that WWTPs play a critical role in safeguarding public health and protecting the environment by removing pollutants and pathogens from wastewater before it is discharged into water bodies. Microorganisms, in form of activated sludge, are crucial to the treatment of wastewater, by removing organic and inorganic chemicals (Küster and Adler 2014). Within the activated sludge system, diverse communities of mostly bacteria work synergistically to degrade organic matter, remove nutrients, and purify water (Lee, Kang, and Park 2015). This complex microbial ecosystem forms the backbone of modern WWTPs, highlighting the significance of microbial technology in bioremediation of PPCPs. However, the efficacy of pollutant removal in WWTPs is contingent to the technologies utilised and upon the nature of the compounds to be removed (Margot et al. 2015). While conventional WWTPs may effectively handle hydrophobic pollutants through sorption, PPCPs of hydrophilic nature and those recalcitrant to biodegradation pose a challenge. Chemical composition can also affect biological removal. For instance, compounds with ether bonds (e.g., Methyl tert‐butyl ether), highly branched quaternary carbons (e.g., nonylphenols and alkyl sulfonates), highly halogenated (e.g., chlorinated aliphatic hydrocarbons and perfluorinated compounds), and seven‐membered ring (carbamazepine) are recalcitrant to biodegradation. One of most recalcitrant compounds are halogenated APIs such as diclofenac for which the WWTP removal efficiency is around 21%–40% or compounds having benzene rings, such as carbamazepine, that has two benzene rings fused to an azepine group, for which the removal is below 10% (Zhang, Geißen, and Gal 2008). The prevalence of PPCPs is sewage can also be explained by widespread consumption. This is the case of paracetamol and caffeine (Khasawneh and Palaniandy 2021; Wu, Zhang, and Chen 2012). Additionally, drugs for analgesia (e.g., diclofenac), neurological diseases (e.g., carbamazepine), heart failure (e.g., atenolol), and antibiotics (e.g., sulfamethoxazole (SMX)) contribute to chemical pollution because they are only partially metabolised by the human body (Luo et al. 2014).

A ranking of the most abundant PPCPs is hard to establish due to the fluctuation of the concentration of these compounds in the influents of WWTPs (Aus der Beek et al. 2016; Di Marcantonio et al. 2020). PPCP concentration in effluents also exhibit variability due to seasonal changes correlated with the seasonal consumption of PPCPs and with climatic influence in PPCPs' degradation (Di Marcantonio et al. 2020; Gomez et al. 2007). In the North of China, it was shown that removal is reduced in winter (Li et al. 2018), which highlights the effect of season. An additional example of this variability is a study conducted in Brazil, in which ibuprofen concentrations reached 1.75 μg/L in June, while it was detected at a concentration of 0.006 μg/L in September (Pivetta and Do Carmo Cauduro Gastaldini 2019). Furthermore, a supplementary reason for the inefficiency in the removal of PPCPs in WWTP is their low concentration (ng/L‐μg/L) (Wang et al. 2021; Pivetta and Do Carmo Cauduro Gastaldini 2019). All this results in poor removal of recalcitrant PPCPs in treated wastewater (Larsson and Flach 2022).

Insufficient infrastructure and limited analytical studies in certain areas of the world contribute to an underestimation of pharmaceutical pollution associated with WWTPs (Aus der Beek et al. 2016; Rich, Zumstein, and Helbling 2022; Yan et al. 2023). Excessive rainwater overflow in combined sewer systems result in an upsurge of untreated water discharge in surface water leading to elevated levels of PPCPs in the environment. For instance, concentrations of 14 μg/L of ibuprofen or 1.8 μg/L of diclofenac were observed in the effluents of an overflown WWTP in West Yorkshire (UK) (Kay et al. 2017). Moreover, many countries lack the infrastructure needed to treat wastewater, resulting in direct discharge of PPCPs and their degradation products in water bodies. The concentration of PPCPs can reach, in some cases mg/L, as it was the case for ibuprofen detected in stream water in South Africa (Patrolecco et al. 2013) and diclofenac and triclosan detected in Nigeria and South Africa, respectively (Ajibola et al. 2021; Patrolecco et al. 2013).

Moreover, WWTPs collecting wastewater from pharmaceutical production sites might contribute disproportionally to environmental pollution since they are not able to remove the highly concentrated PPCPs efficiently. For example, ciprofloxacin was found at concentration of 31 mg/L in the WWTP effluents in the proximity of a pharmacological industry in Hyderabab (India) (Larsson, de Pedro, and Paxeus 2007) and naproxen reached 34 mg/L in the effluents of a WWTP in the USA (Kibuye et al. 2019).

Advanced technologies, such as UV, the Fenton process, and ozonation, can support conventional treatment in increasing the efficiency in the removal of PPCPs. However, they potentially yield harmful intermediates despite achieving high removal efficiency for the parent compound (Lado Ribeiro et al. 2019). For example, in some reactions, byproducts like specific carboxylic acids are not easily degraded by ozone, and oxidising radicals are generated, such as hydroxyl radicals (Lado Ribeiro et al. 2019). The effectiveness of granular activated carbon (GAC) and powdered activated carbon (PAC) depends on the chemical properties of the compounds and the organic load present in the treated wastewater that can compete with micropollutants for sorption surface. Moreover, ozonation, coagulation, and filtrations are less efficient when applied to micropollutant removal in comparison to the afore‐mentioned techniques (Belete et al. 2023).

## 6 The Role of Biological Strategies in PPCP Removal Applied in Wastewater

Advanced biological techniques (e.g., membrane bioreactors (MBR), moving bed biofilm reactors (MBBR), or upflow anaerobic sludge blanket (UASB)) can be implemented in WWTPs to use microorganisms to degrade organic pollutants and effectively reduce the biochemical oxygen demand (BOD) (Taheran et al. 2016). The membrane allows for an extended Sludge Retention Time (SRT) (Lado Ribeiro et al. 2019; Margot et al. 2015) that favours the colonisation by slowly growing degraders (Taheran et al. 2016). The membrane favours colonisation by a more diverse microbial community, which has a broader potential for PCPP removal, since it enables the retention of slow‐growing microorganisms such as nitrifiers (Clara et al. 2005). The nitrifying bacteria have been described as involved in the co‐metabolic degradation of target pollutants owing to the production of promiscuous ammonia monooxygenases (Belete et al. 2023; Cirja et al. 2008; Guerra et al. 2014; Luo et al. 2014). A lower hydraulic retention time (HRT) could enhance the removal capabilities of WWTP. This has been shown in a study where an HRT of 16 h was crucial to increase the microbiological removal of PCPPs for most of the analgesics (e.g., ibuprofen) (Guerra et al. 2014). The utilisation of a sludge carrier with suspended biofilm improves the removal of specific micropollutants (Falås et al. 2012, 2013). This approach enhances the degradation or transformation of substances such as diclofenac, ketoprofen, and mefenamic acid (Falås et al. 2012). The introduction of additional nutrients (biostimulation) in a moving bed biofilm reactor (MBBR) has also been shown to enhance effectiveness. This has been reported in two studies implementing this approach to increase the removal efficiency of PPCPs in effluents. The first study demonstrated a positive effect of elevated chemical oxygen demand (COD) and ammonia addition, favouring the degradation of micropollutants and stimulating co‐metabolism. This effect was particularly pronounced when conditions were optimised for specific groups of compounds, leading to improved removal of atenolol and diclofenac (Tang, Rosborg, et al. 2021). A second study with synthetic wastewater compared the addition of oxidising agent such as manganese to a MBBR resulting in an enhanced degradation rate for most compounds. In the presence of manganese‐oxidising bacteria, manganese has an even higher oxidative potential than in abiotic controls, favouring the removal of micropollutants such as EDCs and sulfonamides (Wang et al. 2022). Notably, the degradation of diclofenac showed a sevenfold improvement compared to the control MBBR without additional feeding (Wang et al. 2022).

Hybrid systems integrating advanced biological methods coupled with physical ones can be more impactful. UASB complemented with Fenton or photo‐Fenton reactions resulted in the removal of 99.9% of organic pollutants (trazine, rifampicin, and EE2) at a concentration around 350 μg/L (Rodrigues‐Silva et al. 2022). Moreover, MBR coupled with UV oxidation, activated sludge, and gamma irradiation has a good impact on PPCPs removal. More than 95% of endocrine‐disruptive chemicals, ß‐blockers, analgesics, at a concentration of 5 μg/L were removed from synthetic wastewater in such a system (Dhangar and Kumar 2020). A study showed that MBR coupled to reverse osmosis ensures 99% removal of the target compounds (Dhangar and Kumar 2020).

The results of the studies above clearly underscore the importance of biological treatment methods and the need to be further studying those for their implementation at a larger scale. Consequently, the forthcoming sections focus on examining the specific mechanisms and effectiveness of biological mechanisms in detail.

## 7 Bioremediation of PPCPs by Microbial Strains and Communities

In the intricate landscape of PPCPs degradation, certain microbial players have consistently emerged as central figures. These microorganisms play a key role in environmental remediation and biotransformation processes of multiple pollutants. Whether in soil, water, or other ecological niches, these microbial players exhibit remarkable adaptability and resilience, effectively metabolising pharmaceutical residues. Moreover, in recent years, microbial communities and single strains have been tested at lab‐scale to assess the removal capabilities of PPCPs (Table 2). *Rhizobiales, Burkholderiales, and Actinomycetales* are associated with improved water quality in WWTPs (Fan et al. 2017). Single strains are known to be able to remove efficiently high concentration of pharmaceuticals. Bacteria such as *Variovorax* (Murdoch and Hay 2015) and *Sphingobium yanoikuyae* (Balciunas et al. 2020) are involved in ibuprofen degradation, *Nocardia europea* in triclosan degradation (Roh et al. 2009), and *Acinetobacter* (Wang, Hu, and Wang et al. 2018) and *Microbacterium* (Ricken et al. 2017) in SMX degradation. Some genera have the capacity to degrade several PPCPs. Various *Pseudomonas* strains can degrade acetaminophen, caffeine, EE2, and carbamazepine (Li et al. 2013; Park and Oh 2020; Rios‐Miguel et al. 2022; Sabirova et al. 2008), while various *Bacillus* strains can degrade naproxen, acetaminophen, trimethoprim, and sulfamethoxazole. Moreover, some bacteria mineralize PPCPs, using them as sole nitrogen or carbon source, which is advantageous because less additional nutrients are needed for allowing bacterial growth. *Achromobacter* was able to grow on SMX as sole nitrogen source and *Rhizobium* C12 was able to grow on carbamazepine as sole carbon source (Bessa et al. 2017). Another strategy is applying thermophiles, which have a promising enzymatic machinery for xenobiotic transformation. However, their cultivation demands higher temperatures. *Thermus thermophilus* has been used to degrade recalcitrant compounds such as ciprofloxacin antibiotics, reaching 57% removal at 70°C (Pan et al. 2018). In other cases, the degradation process can even result in the production of energy, as reported in a photolytic MBR (Sogani et al. 2021). Here, *Rhodopseudomonas palustris* was efficient in degrading ethinylestradiol, while simultaneously generating energy. Even though using pure single strains are unrealistically applied in a real case bioremediation, they still can be used for bioaugmenting a natural community. *Sphingobium yanoikuyae* has been applied to wetlands to enhance the removal efficiency of ibuprofen contaminated water by *Juncus effusus* (Balciunas et al. 2020).

**TABLE 2:** Microbial pure strains and microbial communities known capable of removing pollutants.

Organisms/community	Conditions	Pharmaceutical	Quantity (mg/L)	Degradation (%)	Time	Ref.
*Bacillus thuringensis* B1	pH 6.5 30°C Co‐metabolism with glucose (0.5 g/L)	Naproxen	12	100	Approx 20 days	Górny et al. (2019)
*Planococcus* sp.	Co‐metabolism (phenol) Mineral media 30°C	Naproxen	6	86	35 days	Domaradzka et al. (2015)
*Pseudoxanthomonas sp*. DIN‐3	30°C	Naproxen	45	55.3	7 days	Lu et al. (2019)
Consortium‐ 6 generation adapted	MSM	Ibuprofen	50	100	12 h	Chen et al. (2023)
Consortium	MSM	Ibuprofen	500	100	28 h	Aguilar‐Romero et al. (2024)
*Pseudomonas alloputida*	MSM	Ibuprofen	20	83	24 h	Chen et al. (2023)
*Sphingobium yanoikuyae*	Mineral Media	Ibuprofen	500	100	50 h	Balciunas et al. (2020)
*Variovorax* Ibu‐1	MSM liquid	Ibuprofen	500	> 95	> 150 h	Murdoch and Hay (2015)
*Patulibacter sp*. I11	OD2‐medium	Ibuprofen	50	92	70 h	Almeida et al. (2013)
Activated sludge	Anaerobic reactor 35°C	Diclofenac	120 (mg/kg TSS)	30	24 days	Yang et al. (2022)
Activated sludge	Raw municipal water	Acetaminophen	50	100	48 h	Palma et al. (2018)
*Pseudomonas* spp.^a^	Synthetic media	Acetaminophen	200	100	10 h	Park and Oh (2020), Rios‐Miguel et al. (2022)
*Pseudomonas moorei* KB4	MSM	Acetaminophen	50	100	24 h	Żur, Wojcieszyńska, et al. (2018)
*Bacillus cereus*	MSM	Acetaminophen	200	100	144 h	Palma, Magno, and Costa (2021)
Co‐culture of *Stenotrophomonas* and *Pseudomonas*	MSM	Acetaminophen	2000	100	35 h	Zhang et al. (2013)
*Hydrogenophaga sp*.	1/10 ST medium, 5 mg/L yeast extract, 50 mg/L peptone	Atenolol	0.300	82.2	72 h	Yi et al. (2022)
Acclimatised activated sludge	SBR, GRT: 40 h	Atenolol	400	91	—	Rezaei, Aghapour, and Khorsandi (2022)
Acclimatised activated sludge	10% PBS	SMX	100	100	12 h	He et al. (2024)
*Sphingobacterium mizutaii*	MSM 30°C	SMX	50	93.7	7 days	He et al. (2024), Song et al. (2021)
*Achromobacter* sp. JL9	As nitrogen source with extra carbon source	SMX	50	63.09	120 h	Liang and Hu (2019)
Colonised zeolite filter	—	SMX	0.003	45	—	Cuomo et al. (2024)
*Rhodococcus equi*	Glucose 26°C MMSM	SMX	6	29	120 h	Larcher and Yargeau (2011)
*Sphingobium mizutaii*	30°C MSM	SMX	50	93.87	7 days	Song et al. (2021)
*Acinetobacter* sp.	25°C	SMX	30	100	5 h	Wang, Hu, and Wang (2018)
Adapted Activated Sludge	MM Trace element	SMX	160	96		Wang and Wang (2018)
*Microbacterium* sp. Strain BR1	Artificial urine	SMX	253	100	48 h	Ricken et al. (2017)
*Bacillus subtilis*	Inorganic solid media 5% yeast powder	Trimetropim+SMX	5	70	10 days	Liu et al. (2018)
*Pseudomonas putida* CBB5	YNB‐supplemented medium	Caffeine	2500	100	53 h	Chi et al. (2009)
*Psuedomonas alcaligenes* CFR 1708	CLM	Caffeine	1000	100	> 4 h	Babu et al. (2005)
*Kleisbella* and *Rhodococcus* as mix culture	Salt medium 30°C Resting cells	Caffeine	1000	100	10 h	Madyastha and Sridhar (1998)
*Rhodopseudomonas palustris*	Photobioreactor Anaerobic Glycerol	EE2	1	72	16 days	Sogani et al. (2021)
*Nitrosomonas europea*	pH 7 30°C Modified medium B	EE2	0.4	100	180 h	Shi et al. (2004)
Nitrifying activated sludge	pH 7 30°C	EE2	1	> 90	144 h	Shi et al. (2004)
*Rhodococcus erythropolis*	Extra carbon source 26°C MMSM pH 7 yeast extract	EE2	1.4	47	13 h	O'Grady, Evangelista, and Yargeau (2009)
*Sphingobacterium* sp. JCR5	30°C MM	EE2	30	87	10 days	Haiyan et al. (2007)
*Pseudomonas putida*	50 μM Mn^2+^ Glucose	EE2	Trace concentration: 0.148	100	72 h	Sabirova et al. (2008)
*Sphingopyx* strain KCY1	Nitrate mineral salts	Triclosan	5	100	2 days	Lee et al. (2012)
Adapted Consortium	MSM	Triclosan	500	100	14 days	Hay, Dees, and Sayler (2006)
*Nocardia europea*	Acetone free media	Triclosan	2.5	100	90 h	Roh et al. (2009)
Communities growing in anaerobic condition	Anaerobic Mineral media Yeast extract 40 mM methanol 20 mM sulfate	Ciprofloxacin	0.5	85	6 days	Martins, Sanches, and Pereira (2018)
*Labrys portucalensis*	25°C Acetate Mineral media	Ciprofloxacin	1.2	85	28 days	Amorim et al. (2014)
Bacteria mixed culture	30°C Mineral media Carbon source	Ciprofloxacin	10	61.5	14 days	Feng et al. (2019)
*Ochrobactrum* sp. YJ17	30°C Mineral media Carbon source	Ciprofloxacin	10	34.3	14 days	Feng et al. (2019)
*Thermus thermophilus*	70°C pH 6.5 Malt extract	Ciprofloxacin	5	57	5 days	Pan et al. (2018)
*Rhodococcus rhodochronous*	Brain infusion media Glucose 3 g/L	Carbamazepine	12	15	28 days	Gauthier, Yargeau, and Cooper (2010)
*Psuedomonas* sp. CBZ 4	pH 7 10°C	Carbamazepine	100	46.6	144 h	Li et al. (2013)
*Paraburkholderia xenovorans* LB400	Mineral media 28°C Yeast extact	Carbamazepine	10	100	24 h	Aukema et al. (2017)
*Starkeya* sp.*/Rhizobium sp*.	25°C MM	Carbamazepine	10	35	15 days	Bessa et al. (2017)
Mixed microbial culture	LB 30°C	Carbamazepine	0.1	60	12 days	Ha et al. (2016)

Another efficient strategy is to use microbial communities for the biodegradation of pollutants (Table 2). The beneficial effect of bioaugmentation on ibuprofen removal by applying adapted microbial communities was shown in laboratory‐scale experiments (Aguilar‐Romero et al. 2024). In this setting, natural communities were enriched with an adapted consortium of ibuprofen‐degrading microbes, belonging to the genera *Pseudomonas*, *Sphingomonas*, and *Achromobacter*. The achieved ibuprofen removal rate reached 90%, compared to only 30% removal in the non‐bioaugmented system. The fact that adapted microbial communities can produce a broad variety of efficient enzymes that can act synergistically to tackle pollution (De Roy et al. 2014) can explain this improved performance. When certain compounds undergo catabolic processes, they can lead to the formation of dead‐end products, which can be utilised farther by microbial communities when working collaboratively. For instance, the microbial consortium consisting of *Paenarthrobacter* and *Achromobacter* demonstrated the capability to degrade SMX, resulting in the excretion of the dead‐end product 3‐amino‐5‐methylisoxazole (3A5MI) in the medium. This product was subsequently removed when the simplified community was enriched with *Nocardioides*, *Chryseobacterium*, *Acidovorax*, and *Sphingobium* (Qi et al. 2021).

Moreover, bacterial communities, with their diverse genetic makeup and cooperative interactions, are effective for bioremediation purposes (Aguilar‐Romero et al. 2024; Chen et al. 2023; Ha et al. 2016; Hay, Dees, and Sayler 2006; He et al. 2024). Based on the data presented in Table 2, an efficient removal of certain PPCPs can be achieved by either a natural community, such as activated sludge, or a laboratory‐adapted community containing genera like *Pseudomonas*, *Sphingomonas*, *Achromobacter*, and *Methylobacillus*. Those PPCPs include ibuprofen (Aguilar‐Romero et al. 2024; Chen et al. 2023), atenolol (Rezaei, Aghapour, and Khorsandi 2022), SMX (He et al. 2024), EE2 (Shi et al. 2004), and triclosan (Hay, Dees, and Sayler 2006). Moreover, the use of a community for removal can be more straightforward since the isolation of single strains from a community is not always possible. In some cases, biofilm formation on a physical growth support can help to consolidate the microbial community. This was shown in a study in which the removal of SMX was faster and more efficient in a zeolite filter colonised by *Caulobacterales*, *Rhizobiales*, and *Burkholderiales* in comparison to degradation with no filter (55% versus 10% SMX removal, respectively) (Cuomo et al. 2024). Lab‐adapted communities represent a successful strategy for targeting a wide range of PPCPs. For instance, feeding lab‐scale MBRs with a mixture of PPCPs without excess sludge removal applied selective pressure on bacterial communities consisting mainly of *Achromobacter*, *Cupriavidus*, and *Pseudomonas*. Due to the system's extended sludge retention time, this resulted in significant removal rates ranging from 75% to 100% for the pollutants applied to the MBRs (Suleiman et al. 2023).

Biostimulation is another valuable approach to improve the degradative capabilities of microbial communities. For example, MBR microbial communities removed significantly recalcitrant pollutants, such as ibuprofen and enalapril only when caffeine and acetaminophen were added in the influent. This highlights that the background pollution profile is crucial for an efficient removal of specific pollutants (Suleiman et al. 2024). Some studies have shown that anaerobic processes can effectively remove persistent micropollutants, since some reactions, such as dehalogenation and reduction of nitro groups occur under anaerobic conditions. For instance, ciprofloxacin was removed at a rate of 80% under nitrate and sulfate reduction conditions by a microbial community composed mainly of *Desulfovibrio*, *Enterococcus*, and *Peptostreeptococcus* (Martins, Sanches, and Pereira 2018). In the same manner, a 30% removal of diclofenac was successfully achieved when activated sludge was cultivated under anaerobic conditions. It was proposed that *Proteiniclasticum* and *Tissierellales* were involved in diclofenac removal in this bacterial community (Yang et al. 2022).

## 8 Enzymes Involved on PPCP Degradation

Enzymes play a crucial role in the biodegradation of pollutants, serving as nature's biochemical scissors to break down harmful substances into simpler, less harmful compounds. These powerful biocatalysts are produced by single strains or microbial communities in various environments where pollution occurs. In certain instances, they are involved in the complete mineralization of pollutants with concomitant production of energy and growth. Enzymes can function in a promiscuous (large spectrum of substrates) or specific way (narrow spectrum of substrate). Hydroxylation, hydrolysis, methylation, acetylation, and oxidoreduction of PPCPs are common promiscuous reactions involved in their degradation (Kolvenbach et al. 2014; Stadlmair et al. 2018). One example of promiscuous enzymes known for the degradation of paracetamol is amidases (Rios‐Miguel et al. 2022), which are also supposedly involved in the first step of the degradation of atenolol (Xu et al. 2017) and carbamazepine, followed in their action by dioxygenases (Wang et al. 2023). A dioxygenase activity has been shown in cell extracts of *Sphingopyxis* KCY1, able to degrade triclosan (Lee et al. 2012). Moreover, it is hypothesised that hydroxylating enzymes play a crucial role in acetaminophen and ibuprofen biodegradation (Żur, Piński, et al. 2018).

The elimination of PPCPs in WWTPs is related to the production of hydroxylating enzymes so called monooxygenases, such as the ammonia monooxygenases (AMO), that degrades xenobiotic compounds in non‐specific manner (Fernandez‐Fontaina et al. 2016; Tang, Rosborg, et al. 2021; Xu et al. 2017; Yi and Harper 2007). The prevalence of ammonia‐oxidizers (and thus AMO) in the presence of ammonia facilitates the degradation of EE2 (Yi and Harper 2007) and of atenolol (Xu et al. 2017). A cytochrome P450 monooxygenase was also identified as contributing to ciprofloxacin biodegradation facilitating the degradation of these recalcitrant compounds (Jia et al. 2018). It was shown that activated sludge in a MBR catalyses the transformation of diclofenac into 4′‐hydroxydiclofenac similarly to the action of the cytochrome P450 monooxygenase (Bouju et al. 2016). Aliphatic and hydroquinone monooxygenases are possibly involved in the degradation pathway of ibuprofen via the formation of hydroxy‐ibuprofen (Żur, Piński, et al. 2018). Naproxen degradation by *Planococcus* and *Amycolatopsis* is mainly attributed to monooxygenases and dioxygenases (Alanis‐Sánchez et al. 2019; Wojcieszyńska et al. 2016). Other monooxygenases that act on specific PPCPs are SadA and SadB, which are involved in the transformation of SMX into its dead‐end metabolite 3A5MI (Reis et al. 2019). A 9α‐monooxygenase isolated from *Rhodococcus* was involved in the conversion of EE2 into its hydroxylated metabolite (Larcher and Yargeau 2013). Specific enzymes play crucial roles in both the synthesis and degradation of caffeine. Examples include caffeine dehydrogenases, encoded by *cdh* genes, and N‐demethylases genes cluster encoded by *ndm*, which contain dioxygenases, oxidoreductases, and glutathione S‐transferases. These enzymes facilitate the transformation of caffeine into compounds like theobromine and para‐bromine (Lin et al. 2023; Rich, Zumstein, and Helbling 2022).

## 9 Conclusions

The ineffective removal of PPCPs during traditional wastewater treatment represents a growing threat to environmental and public health. The persistence of these contaminants in aquatic ecosystems not only leads to contamination of water bodies and bioaccumulation in food, but also drives the spread of ARGs and endocrine disruptors, which pose significant risks to human health. Addressing this issue requires a holistic “One Health” approach, which integrates the interconnectedness of human, animal, and environmental health. Including additional steps of microbial bioremediation during wastewater treatment offers a promising solution within this framework, allowing the harnessing of microbial metabolism to degrade or neutralise PPCPs. The adaptability and resilience of microbial communities to various pollutants make them a sustainable, cost‐effective alternative to traditional water treatment methods. Furthermore, incorporating advanced techniques like bioaugmentation, microbial consortia engineering, and enzyme‐mediated degradation can enhance the efficacy of microorganism‐based treatments.

By integrating microbial bioremediation in the One Health approach, we can develop a comprehensive strategy to mitigate PPCP contamination, protect ecosystem integrity, and promote public health, highlighting the need for continued research and cross‐disciplinary collaboration.

## Author Contributions

**Francesca Demaria:** writing – original draft, writing – review and editing. **Marcel Suleiman:** writing – review and editing. **Philippe Corvini:** conceptualization, investigation, resources, writing – original draft, writing – review and editing. **Pilar Junier:** writing – review and editing.

## Conflicts of Interest

The authors declare no conflicts of interest.

## Data Availability

Data sharing not applicable to this article as no datasets were generated or analysed during the current study.
